# Supplementary material for: Archaeobotanical and chemical investigations on wine amphorae from San Felice Circeo (Italy) shed light on grape beverages at the Roman time
Source: PLoS One. 2022 Jun 29;17(6):e0267129. doi: 10.1371/journal.pone.0267129 (PMC9242518; doi:10.1371/journal.pone.0267129)
Supplement: S1 Fig — ESEM pictures of Vitis vinifera pollen grains recovered from (A) grapefruits from wild grapes in Tivoli and (B) Fossil sediment from Rignano Flaminio. Pollen grains are tricolpate, ranging from 18–27 μm, with micro-rugulate ornamentation. Unlike SEM references for Vitaceae [49], no porus was observed along the colpi. (DOCX) [file pone.0267129.s001.docx]

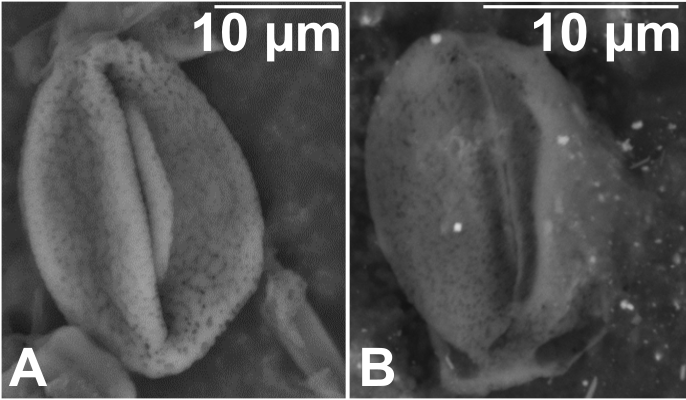


**S1 Fig. ESEM pictures of *Vitis vinifera* pollen grains recovered from (A) grapefruits from wild grapes in Tivoli and (B) Fossil sediment from Rignano Flaminio** Pollen grains are tricolpate, ranging from 18-27 µm, with micro-rugulate ornamentation. Unlike SEM references for Vitaceae (49), no porus was observed along the colpi.
