# Supplementary material for: Archaeobotanical and chemical investigations on wine amphorae from San Felice Circeo (Italy) shed light on grape beverages at the Roman time
Source: PLoS One. 2022 Jun 29;17(6):e0267129. doi: 10.1371/journal.pone.0267129 (PMC9242518; doi:10.1371/journal.pone.0267129)
Supplement: Attachment — Submitted filename: Response to reviewer R2.docx [file pone.0267129.s002.docx]

**Response to the editor**

We would like to thank the editor for the helpful comment he made on the funding statement and the acknowledgement.

We removed the funding statement in the acknowledgments and left it as it initially stood in the funding acquisition part.

The latest version of the manuscript file was uploaded as the main article file, without track changes or highlighting.

Regarding the comment for the data availability statement,

The data availability statement was updated as follows:

All the data related to pollen and chemical analysis are within the main text of the manuscript (in the table 1 and figures).

Archaeobotanical pictures (pollen and vegetal tissues) are shown in the figures.

Chemical information is shown in the chromatogram in the figures and the main text.

The Supporting Information files includes two pictures of SEM microscopy showing morphological features of Vitis already described in the main text.

No other data were collected.

**Response to the reviewer**

Additionally, we would like to thank the reviewer for the important comments he reported in the manuscript. In red, we answered and detailed the modifications we proposed.

Reviewers' comments:

Reviewer's Responses to Questions

**Comments to the Author**

1. Is the manuscript technically sound, and do the data support the conclusions?

The manuscript must describe a technically sound piece of scientific research with data that supports the conclusions. Experiments must have been conducted rigorously, with appropriate controls, replication, and sample sizes. The conclusions must be drawn appropriately based on the data presented.

Reviewer #1: **Partly**

2. Has the statistical analysis been performed appropriately and rigorously?

Reviewer #1: Yes

3. Have the authors made all data underlying the findings in their manuscript fully available?

The [PLOS Data policy](http://www.plosone.org/static/policies.action#sharing) requires authors to make all data underlying the findings described in their manuscript fully available without restriction, with rare exception (please refer to the Data Availability Statement in the manuscript PDF file). The data should be provided as part of the manuscript or its supporting information, or deposited to a public repository. For example, in addition to summary statistics, the data points behind means, medians and variance measures should be available. If there are restrictions on publicly sharing data—e.g. participant privacy or use of data from a third party—those must be specified.

Reviewer #1: Yes

4. Is the manuscript presented in an intelligible fashion and written in standard English?

PLOS ONE does not copyedit accepted manuscripts, so the language in submitted articles must be clear, correct, and unambiguous. Any typographical or grammatical errors should be corrected at revision, so please note any specific errors here.

Reviewer #1: Yes

5. Review Comments to the Author

Reviewer #1: The manuscript presents a study of Roman wine amphorae combining chemical palaeobotanical analyses. It provides interesting insights and leads to original hypotheses about the grape juice beverages that may have been contained in the amphorae and about the vines that produced them.

My advice is to publish the article with minor changes. The article could be shortened a bit and presented in a more concise form. It contains a large number of bibliographical references.

Considering your remark, this is true that the publication includes a large number of bibliographical references. However, the present paper aims at combining 3 different disciplines (archaeology, botanical analysis and organic residue / chemical analysis). The number of citations required is therefore elevated to provide a concrete and well-documented work.

The « Materials and Methods » section is lengthy and contains details that do not seem necessary, in particular in the « **Archaeological context** » and « **Solvents** » sections.

The “Solvents” section was entirely removed.

The sections “Sample preparation for chromatographic analyses” and “Gas chromatography – Mass Spectrometry” were combined. The complete extractive protocol had been recently published; hence the citation was uploaded.

The “Archaeological context” section remains fundamental for the well-understanding of the article, specially for the archaeological consideration of the site to put in perspective the archaeobotanical results. Moreover, the archaeological details consigned in the introduction are helpful to understand the results obtained. Therefore, it seems complicated to us to shorten this section without detracting from the overall understanding of the article and the archaeobotanical results.

The introduction is also a bit long and mainly takes the form of a historiographical presentation of the various chemical and palynological analyses of amphora contents carried out so far. For the sake of the article it would be better to have a slightly more concise introduction, not focusing so much on the methodological angle but highlighting the questions addressed by the study of the type of material and by this specific study.

Considering your comment, the introduction was slightly shortened. However, the introduction is less than 160 words and aimed at detailing all of the 3 disciplines considered in the research article. A particular interest was given to highlight the interdisciplinary and innovative character from a methodological point of view since such combined studies have been rarely conducted. In these conditions, it remains complicated to shorten more the introduction without impacting its integrity.

Following the PlosOne journal recommendation for the introduction, we provided the background context with a methodological angle before naming the purpose and the significance of the study. We carefully developed the problematic regarding the urging need of interdisciplinary methods to understand archaeological objects to prevent overinterpretation or misinterpretation raised by single method results.

One of the most innovative aspects of the article certainly concerns the observations on Vitis pollens and the hypotheses that are drawn from them on the nature and origin of the vines used for the production of the beverages contained in the amphorae.
However this aspect of the article deserves to be clarified.

In section « Pollen » (202) it is a bit difficult to understand which pollen morphotypes can be encountered in modern vines and which are closer to the archaeologicla pollen grains. It would be good to discribe briefly the morphotypes typical of modern wild male flowers, modern wild female flowers and modern hermaphrodite domesticated varieties (female varieties if known).
A sentence was added (L. 367) to summarize the different morphologies encountered.

As it stands I am not convinced at all by your second hypothesis, the use of indigenous cultivars. Contrary to what you say, I do not think that the data you present strongly support this hypothesis. You list several indigenous female varieties. Such varieties are indeed in the minority, but they can nevertheless be found in various regions and are not specifically local.

We thank the reviewer for tis comment which gives us the opportunity to better explain the second hypothesis. We added a sentence as suggested (L. 384).

The resemblance to local Pleistocene pollens does not seem to me to be a valid argument either; or Pleistocene or early Holocene pollens from other regions should be shown to have a different morphology. If I have misunderstood your argument, I apologise and please clarify your thinking. Otherwise I think that this second hypothesis should be abandoned.

Reading again our second hypothesis, it is true that it was not explained clearly enough. For this reason, we made changes throughout the paragraph to mitigate the interpretation. It remains important for us to keep this hypothesis as one of the possible interpretations to the understanding of the tricolpate pollen in order to leave open a discussion on the subject since this pollen has not been reported in these conditions.

**Minor comments**

- Table 1 ; I don’t see how you could have 1.6% Erica from a total of 31 pollen grains. Please check all values in Table 1.

After revision, the total pollen grain for the amphora SFC2 is 61 instead of 31. Percentages were recalcultated.

- 305. Please replace « in this area since at least » by « in this area during the Middle Pleistocene ». Or else provide evidence that wild grapevine was recorded since the Middle Pleistocene.

Modification done.

- 384-407. Third hypothesis. I would guess that Pliny refers to male wildflowers. Can you confirm that or do you have more specific information on that matter?

Pliny the elder did not specifically mention “male wildflower” but we can assume he was talking about such flower since he never mentioned the female wildflower in his Naturalis historia.

6. PLOS authors have the option to publish the peer review history of their article ([what does this mean?](https://journals.plos.org/plosone/s/editorial-and-peer-review-process#loc-peer-review-history)). If published, this will include your full peer review and any attached files.

If you choose “no”, your identity will remain anonymous but your review may still be made public.

**Do you want your identity to be public for this peer review?** For information about this choice, including consent withdrawal, please see our [Privacy Policy](https://www.plos.org/privacy-policy).

Reviewer #1: No
